# Variation in Effects of Non-Hodgkin Lymphoma Risk Factors According to the Human Leukocyte Antigen (*HLA*)-DRB1*01:01 Allele and Ancestral Haplotype 8.1

**DOI:** 10.1371/journal.pone.0026949

**Published:** 2011-11-09

**Authors:** Sophia S. Wang, Yani Lu, Nathaniel Rothman, Amr M. Abdou, James R. Cerhan, Anneclaire De Roos, Scott Davis, Richard K. Severson, Wendy Cozen, Stephen J. Chanock, Leslie Bernstein, Lindsay M. Morton, Patricia Hartge

**Affiliations:** 1 Division of Cancer Etiology, Department of Population Sciences, Beckman Research Institute and City of Hope, Duarte, California, United States of America; 2 Division of Cancer Epidemiology and Genetics, National Cancer Institute, National Institutes of Health (NIH), United States Department of Human Health Services (DHHS), Rockville, Maryland, United States of America; 3 Department of Microbiology and Immunology, National Research Center, Cairo, Egypt; 4 Division of Epidemiology, College of Medicine, Mayo Clinic, Rochester, Minnesota, United States of America; 5 Fred Hutchinson Cancer Research Center, University of Washington, Seattle, Washington, United States of America; 6 Department of Family Medicine and Public Health Sciences, Karmanos Cancer Institute, Wayne State University, Detroit, Michigan, United States of America; 7 Norris Comprehensive Cancer Center, University of Southern California, Los Angeles, California, United States of America; Kings College, London, United Kingdom

## Abstract

Genetic variations in human leukocyte antigens (*HLA*) are critical in host responses to infections, transplantation, and immunological diseases. We previously identified associations with non-Hodgkin lymphoma (NHL) and the HLA-DRB1*01:01 allele and extended ancestral haplotype (AH) 8.1 (HLA-A*01-B*08-DR*03-TNF-308A). To illuminate how *HLA* alleles and haplotypes may influence NHL etiology, we examined potential interactions between HLA-DRB1*01:01 and AH 8.1, and a wide range of NHL risk factors among 685 NHL cases and 646 controls from a United States population-based case-control study. We calculated odds ratios and 95% confidence intervals by *HLA* allele or haplotype status, adjusted for sex, age, race and study center for NHL and two major subtypes using polychotomous unconditional logistic regression models. The previously reported elevation in NHL risk associated with exposures to termite treatment and polychlorinated biphenyls were restricted to individuals who did *not* possess HLA-DRB1*01:01. Previous associations for NHL and DLBCL with decreased sun exposure, higher BMI, and autoimmune conditions were statistically significant only among those *with* AH 8.1, and null among those *without* AH 8.1. Our results suggest that NHL risk factors vary in their association based on HLA-DRB1*01:01 and AH 8.1 status. Our results further suggest that certain NHL risk factors may act through a common mechanism to alter NHL risk. Finally, control participants with either HLA-DRB1*01:01 or AH 8.1 reported having a family history of NHL twice as likely as those who did not have either allele or haplotype, providing the first empirical evidence that *HLA* associations may explain some of the well-established relationship between family history and NHL risk.

## Introduction

Human leukocyte antigens (*HLA*) are among the most polymorphic genes in humans and result in variations of the peptide-binding cleft, influencing the antigens bound and presented to T cells [1]. In general, *HLA* Class I molecules (*HLA-A*, *-B*, and *-C*) present foreign antigens to CD8+ cytotoxic T lymphocytes (CTL), and *HLA* Class II molecules (*HLA-DR*, -*DQ*, and -*DP*) present antigenic peptides to CD4+ T helper cells [2]. *HLA* play critical roles in human immunological diseases, transplantation, and host defense against infections including progression to the acquired immunodeficiency syndrome [3], all known risk factors for non-Hodgkin lymphoma (NHL).

We previously conducted *HLA* Class I (A, B, C) and Class II (DR) genotyping in a population-based multi-center case-control study of NHL in the United States (U.S.). We reported HLA-DRB1*01:01 as a novel susceptibility allele in NHL risk [4], particularly for the follicular lymphoma subtype, which was consistent with results from a genome-wide association study [4]. We also previously reported the association between the pro-inflammatory cytokine, tumor necrosis factor (*TNF*) G-308A promoter polymorphism with NHL and specifically with diffuse large B-cell lymphoma (DLBCL) [5], a finding also confirmed in large consortial efforts [6], [7]. Although we previously evaluated the joint effects between *TNF* G-308A and NHL risk factors [8], we have since demonstrated that the ancestral haplotype 8.1 (AH 8.1: HLA-A*01-B*08-DR*03-TNFG-308A) whereby *HLA* alleles are in linkage disequilibrium with *TNF*, is associated with DLBCL [9]. Notably, all individuals with the HLA-A*01-B*08-DR*03 haplotype also have the variant *TNF* G-308A polymorphism in our population [9]. Because previous investigations have demonstrated elevated TNF-alpha expression among healthy individuals with AH 8.1, it is plausible that the AH 8.1 reflects a more accurate downstream phenotype, reflecting a synergistic relationship between *TNF* and *HLA* and chronic inflammatory status that would result in elevated DLBCL risk [10]. On the other hand, possession of HLA-DRB1*01:01 likely confers a different phenotype as it is associated with rheumatoid arthritis and may reflect propensity for generating autoantibodies.

Confirmed risk factors for NHL include autoimmune conditions [11], certain infectious agents [12], and family history of lymphoma [13]. Suggestive associations also include increased NHL risk with smoking [14], obesity [15], and decreased NHL risk with alcohol intake [16] and sunlight [17]. A growing body of evidence also implicates organochlorine pesticides in NHL risk [18], [19]. Evidence for decreased NHL risk with atopic conditions (e.g., allergies and asthma) and vitamin B6, height, and later birth order has also been reported, but these associations require further replication [20].

To illuminate how *HLA* alleles and haplotypes may influence NHL etiology, we explored potential interactions between implicated *HLA* alleles and haplotypes, specifically HLA-DRB1*01:01 and AH 8.1, and NHL risk factors. Each of the risk factors has previously been independently evaluated and we include those for which associations consistent to the current body of literature were found [8]. Because the major subtypes of NHL differ in their pattern of some risk factors, we also examined the distinctive effects on the two major NHL subtypes, DLBCL and follicular lymphoma.

## Materials and Methods

### Study population

Details have been described elsewhere [5]. In brief, the multi-center National Cancer Institute – Surveillance, Epidemiology and End Results (NCI-SEER) NHL case-control study population comprised 1,321 NHL cases identified in four SEER registries (Iowa; Detroit, MI; Los Angeles, CA; Seattle, WA) aged 20–74 years and newly diagnosed between July 1998–June 2000 [5]. Cases were not known to have HIV infection. 1,057 population controls were identified by random digit dialing (<65 years) and from Medicare eligibility files (≥65 years). The response rate for cases was 59% and 44% for controls. Written informed consent was obtained from each participant prior to interview. All study participants were asked to provide a venous blood or mouthwash buccal cell sample. The present analysis was conducted on study participants who provided blood and for whom *HLA* typing was completed (685 cases, 646 controls). Results were nearly identical when we restricted the analysis to the 610 cases and 555 controls who self-reported to be non-Hispanic Caucasians and from whom sufficient DNA were available for *HLA* allelotyping [4], [9]. Because no association was observed between genotype and race or between race and risk factors evaluated, we present results for all participants to maximize our sample size and power for evaluation of interactions. This study was approved by the following institutional review boards: the Institutional Review Board of the NCI (NCI); the Health Sciences Institutional Review Board (USC); the Human Subjects Division Institutional Review Board (UW); the Fred Hutchinson Cancer Research Center Institutional Review Board (FHCRC); the Wayne State University Division of Research Institutional Review Road (Wayne State); and the University of Iowa Institutional Review Board (Iowa). Written consent was obtained from all participants included in the study.

### Histopathology

Pathology information was derived from abstracted reports by the local diagnosing pathologist. All cases were histologically confirmed and coded according to the International Classification of Diseases for Oncology (ICD), 2^nd^ Edition [21] and updated to the WHO/ICD-O-3. We evaluated risk for NHL and two major histologic subtypes: DLBCL (ICD-O-2: 9680-84, 9688) and follicular lymphoma (9690-91, 9695-98).

### Laboratory methods

As previously described, DNA was extracted using Puregene Autopure DNA extraction kits (Gentra Systems, Minneapolis, MN) [5]. Four-digit *HLA* Class I (A, B, C) and Class II genotyping (*DRB1*) was conducted at NCI-Frederick (Frederick, MD) according to sequence-specific oligonucleotide probe (SSOP) hybridization and sequence-based typing protocols developed by the 13^th^ International Histocompatibility Workshop [22]. *HLA* alleles were defined as presence or absence of the specific allele. *TNF* genotyping was conducted at the National Cancer Institute Core Genotyping Facility (Gaithersburg, MD, USA) using the Taqman (Foster City, CA, USA) platform. *TNF G-308A* was defined by genotype (GG (referent), GA and AA) [5]. HLA haplotypes were determined using ‘FastHap’, which determines haplotypes by expectation maximization (http://home.ncifcrf.gov/ccr/lgd/bioinformatics/index_n.asp) [9]. Agreement for quality control duplicates (n = 100) was more than 99%.

### Questionnaire Risk Factor Data

Methods and details of data collection were previously described [8] and risk factors were categorized as previously reported [8]. All study participants were queried on: (i) family history defined as any first-degree family member having NHL or lymphoma not otherwise specified (yes, no); (ii) history of immune-related disorders (Sjogren's syndrome, lupus, Crohn's disease, ulcerative colitis, rheumatic heart disease, polymyalgia rheumatica, sarcoidosis, multiple sclerosis, uveitis, myasthenia gravis, polymyositis, dermatomyositis, and/or celiac disease) (yes, no); (iii) blood transfusions (yes, no); (iv) number of surgeries since birth (0–6, ≥7); (v) birth order (first/middle-born child, last born); (vi) height (<65, 65–70,71+ inches); (vii) body mass index (BMI) as weight (kg) divided by height (m) squared (<25, 25−<35, 35+ kg/m2); (viii) termite treatment via a detailed history of pesticide use in each residence occupied for at least 2 years since 1970 and whether the termite treatments occurred before or after 1988 when the termiticide chlordane was banned in the United States. To accommodate a large number of questions during the interview, we used a split-sample questionnaire design, with the core questions above queried for all respondents and additional questions for either group A (all black and 50% of non-black participants) or group B (50% of non-black participants). Additional questions included those on: (ix) asthma (yes, no) ; (x) vitamin B6 intake, dichomotomized by the median intake among controls (<0.97, ≥0.97 mg); (xi) smoking status (never, ever); (xii) alcohol intake (<1, ≥1 grams/week); (xiii) sunlight in teens and past 10 years (<14, ≥14 hours/week); eye color (brown, hazel, green/blue); and (xiv) hay fever and other allergies, excluding food allergies (yes, no). We note that for the each of the exposures queried as part of the split-sample, we have systematically compared the subgroup of participants to the overall population and have found them to be comparable [8].

#### Biospecimen-based exposures

alpha-Chlordane and PCB180 were measured from a subset of cases and controls from whom dust samples were collected and analyzed (682 cases, 513 controls) [18], [19], [23] (alpha-chlordane: <60.3, 60.3–5,870 ng/g; PCB180 (0–20.7; >20.7 ng/g). PCB180 and total furans were evaluated in plasma samples in a subset of 100 untreated cases and 100 controls [23]: PCB180 (≤28.7 , >28.7 ng/g lipid), total furans (≤0.057, >0.057 pg/g lipid).

### Statistical Analysis

#### Independence of risk factors

Among controls, we calculated odds ratios (OR) and 95% confidence intervals (95% CI) for each risk factor with the dichotomized genotype, comparing the presence of a variant allele or haplotype with the absence of the allele or haplotype (Tables 1 and 2) using unconditional logistic regression. For ordinal risk factors with at least three values, we calculated the P-trend for a linear model.

**Table 1:** Risk factor distribution (demographics and family/medical history) by HLA-DRB1*01:01 and AH 8.1 (HLA-A*01-B*08-DR*03-TNF-308A) among controls in the NCI-SEER NHL multicenter case-control study (adjusted for age, education, sex, race, study center).

	HLA-DRB1*0101			AH 8.1		
	Absent	Present	OR (95% CI)	Absent	Present	OR (95% CI)
*Select demographics*						
Sex						
Male	283	49	1.00 (reference)	278	38	1.00 (reference)
Female	233	48	1.11 (0.71–1.774)	235	28	0.83 (0.49–1.41)
Race						
White	442	89	1.00 (reference)	442	62	1.00 (reference)
Other/Unknown	74	8	0.52 (0.22–1.19)	71	4	NC
Age (y)						
<45	80	14	1.00 (reference)	79	9	1.00 (reference)
45–64	220	35	0.88 (0.44–1.73)	213	32	1.32 (0.60–2.92)
≥65	216	48	1.12 (0.57–2.19)	221	25	0.88 (0.38–2.02)
P-trend						
Education (years)						
<12	49	8	1.00 (reference)	45	7	1.00 (reference)
12–15	308	63	1.44 (0.63–3.29)	317	39	0.77 (0.32–1.88)
>15	159	26	1.19 (0.49–2.90)	151	20	0.69 (0.26–1.81)
P-trend						
Study site						
Seattle	172	27	1.00 (reference)	159	27	1.00 (reference)
Detroit	54	17	2.29 (1.13–4.66)	58	8	0.92 (0.38–2.23)
Iowa	181	38	1.23 (0.71–2.14)	186	24	0.75 (0.41–1.37)
Los Angeles	109	15	1.04 (0.51–2.12)	110	7	0.44 (0.18–1.08)
*Family and medical history*						
Family history of NHL						
No	482	87	1.00 (reference)	481	57	1.00 (reference)
Yes	14	8	**2.81 (1.12–7.05)**	16	5	2.35 (0.81–6.82)
Autoimmune conditions						
No	487	95	1.00 (reference)	489	61	1.00 (reference)
Yes	29	2	NC	24	5	1.62 (0.58–4.50)
Asthma						
No	245	40	1.00 (reference)	233	33	1.00 (reference)
Yes	34	5	0.88 (0.31–2.5)	31	6	1.27 (0.46–3.52)
Allergy						
No	95	19	1.00 (reference)	97	14	1.00 (reference)
Yes	139	32	1.29 (0.67–2.47)	148	13	0.50 (0.20–1.22)
Surgeries (total number)						
0–6	47	7	1.00 (reference)	45	5	1.00 (reference)
≥7	232	38	0.72 (0.28–1.87)	219	34	1.44 (0.48–4.30)
Transfusion						
No	408	86	1.00 (reference)	412	56	1.00 (reference)
Yes	100	11	**0.44 (0.22–0.88)**	95	9	0.69 (0.32–1.48)
Birth order						
First/Middle	168	25	1.00 (reference)	158	22	1.00 (reference)
Last	48	12	1.75 (0.79–3.89)	49	9	0.98 (0.39–2.42)

Abbreviations: AH 8.1: ancestral haplotype 8.1 (HLA-A*01-B*08-DR*03-TNF-308A); HLA: human leukocyte antigen; NC:not calculated due to n<5 in cell; NCI-SEER: National Cancer Institute Surveillance, Epidemiology & End Results; TNF: tumor necrosis factor.

**Table 2:** Risk factor distribution (anthropometrics, sunlight and environmental exposures) by HLA-DRB1*01:01 and AH 8.1 (HLA-A*01-B*08-DR*03-TNF-308A) among controls in the NCI-SEER NHL multicenter case-control study (adjusted for age, education, sex, race, study center).

	HLA-DRB1*0101			AH 8.1		
	Absent	Present	OR (95% CI)	Absent	Present	OR (95% CI)
*Anthropometrics and diet*						
BMI (kg/m2)						
<25	151	30	1.00 (reference)	151	20	1.00 (reference)
25 to <35	290	55	0.93 (0.56–1.54)	288	39	1.03 (0.57–1.85)
≥35	45	6	0.65 (0.25–1.69)	42	4	NC
P-trend			0.44			0.7
Height (inches)						
<65	123	27	1.00 (reference)	128	13	1.00 (reference)
65–70	250	38	0.79 (0.42–1.47)	237	31	1.43 (0.66–3.08)
≥71	118	26	1.24 (0.54–2.90)	120	19	1.90 (0.70–5.18)
P-trend			0.6			0.2
Smoking status						
Never	95	22	1.00 (reference)	99	11	1.00 (reference)
Ever	118	26	1.01 (0.52–1.96)	125	14	0.98 (0.42–2.31)
Ethanol (grams/week)						
<1	86	18	1.00 (reference)	93	7	1.00 (reference)
≥1	121	28	1.40 (0.67–2.92)	122	18	2.54 (0.98–6.59)
Vitamin B6 (milligrams)						
<0.97	105	19	1.00 (reference)	101	17	1.00 (reference)
≥0.97	102	27	1.50 (0.75–3.00)	114	8	**0.39 (0.16–0.97)**
*Sunlight*						
Sun in teens (hours/week)						
≥14	170	41	1.00 (reference)	183	18	1.00 (reference)
<14	61	10	0.57 (0.26–1.26)	59	9	1.54 (0.63–3.81)
Sun in past 10 years (hours/week)						
≥14	85	29	1.00 (reference)	103	7	1.00 (reference)
<14	147	21	**0.37 (0.18–0.75)**	139	20	2.25 (0.85–5.91)
Eye color						
Brown	73	16	1.00 (reference)	77	7	1.00 (reference)
Hazel	41	9	1.02 (0.39–2.64)	44	5	1.40 (0.41–4.76)
Green/blue	120	26	0.97 (0.46–2.06)	124	15	1.36 (0.53–3.53)
P-trend			0.98			0.6
*Environmental exposures*						
Termite treatment <1988						
Not treated <1988	364	75	1.00 (reference)	366	48	1.00 (reference)
None or DK	76	8	0.55 (0.25–1.23)	72	9	1.06 (0.48–2.34)
≥1	76	14	1.08 (0.55–2.11)	75	9	1.19 (0.53–2.66)
P-trend			0.80			0.70
alpha-Chlordane (dust; ng/g)						
<60.3	210	47	1.00 (reference)	210	31	1.00 (reference)
60.3–5,870	59	5	0.39 (0.14–1.06)	56	3	NC
PCB180 (dust; ng/g)						
0–20.7	208	40	1.00 (reference)	211	23	1.00 (reference)
>20.7	61	12	1.02 (0.49–2.16)	55	11	1.93 (0.82–4.41)
PCB180 (blood; ng/g)						
≤28.7	21	3	1.00 (reference)	19	4	1.00 (reference)
>28.7	56	11	NC	58	8	NC
Total furans (blood; mol/g)						
≤0.057	19	1	1.00 (reference)	19	1	1.00 (reference)
>0.057	58	13	NC	58	11	NC

Abbreviations: AH 8.1: ancestral haplotype 8.1 (HLA-A*01-B*08-DR*03-TNF-308A); HLA: human leukocyte antigen; NC:not calculated due to n<5 in cell; NCI-SEER: National Cancer Institute Surveillance, Epidemiology & End Results; TNF: tumor necrosis factor.

#### Effects of risk factors by genotype and p-interaction

For all NHL cases and for DLBCL and follicular lymphoma, we calculated OR and 95% CI for each risk factor using multivariable polychotomous unconditional logistic regression for case-control comparisons. In general, we chose as the reference group the category carrying the lowest NHL risk. To determine the p-interaction, we conducted the Wald Chi-square test for homogeneity of the associations with risk factors by genotype strata. All analyses were conducted using SAS 9.2 (SAS Institute).

Although we calculated p-interaction, we evaluated our results based on comparison to previously known and demonstrated risk factors associations for NHL. Because statistically significant p-interactions can be generated based on risk estimates that go in different directions, our criteria for identifying notable results was equally based on determining whether statistically significant associations were observed and restricted to a specific *HLA* allele or genotype and null in the other allele or genotype, even if the p-interaction was not statistically significant.

## Results

### Associations among controls (Tables 1 and 2)

Among control participants, there was a two-fold association between those with either HLA-DRB1*01:01 or AH 8.1 and reporting having a family history of NHL, compared to those who did not have either allele or haplotype. The risk estimates for family history of NHL were generally similar but statistically significant for HLA-DRB1*01:01 (OR = 2.81, 95% CI = 1.12–7.05) but not AH 8.1 (OR = 2.35, 95% CI = 0.81–6.82). Transfusion history was inversely associated with both HLA-DRB1*01:01 (OR = 0.44, 95% CI = 0.22–0.88) and AH 8.1 (OR = 0.69, 95% CI = 0.32–1.48). Other risk factor associations among controls included vitamin B6 intake with AH 8.1 only and sun in past 10 years with HLA-DRB1*01:01 only. No other risk factors were associated with either HLA-DRB1*01:01 or AH 8.1.

### Effects stratified by HLA-DRB1*01:01 (Tables 3 and 4)

**Table 3:** Association (OR and 95% CI) for NHL, DLBCL, and follicular lymphoma for NHL-relevant risk factors (family/medical history and anthropometrics/diet), by HLA-DRB1*01:01 allele status and adjusted for age, education, sex, race, and study center.

	All NHL					DLBCL					Follicular				
	Absent		Present		P	Absent		Present		P	Absent		Present		P
	N	OR (95% CI)	N	OR (95% CI)		N	OR (95% CI)	N	OR (95% CI)		N	OR (95% CI)	N	OR (95% CI)	
*Family and medical history*															
* Family history of NHL*															
No	499	1.00 (reference)	113	1.00 (reference)		136	1.00 (reference)	30	1.00 (reference)		114	1.00 (reference)	45	1.00 (reference)	
Yes	21	1.11 (0.60–2.06)	3	NC	NC	4	NC	1	NC	NC	5	1.13 (0.42–3.10)	2	NC	NC
Autoimmune conditions															
No	494	1.00 (reference)	115	1.00 (reference)		132	1.00 (reference)	30	1.00 (reference)		113	1.00 (reference)	46	1.00 (reference)	
Yes	32	1.27 (0.76–2.13)	4	NC	NC	9	1.26 (0.58–2.76)	2	NC	NC	7	1.26 (0.53–2.97)	1	NC	NC
Asthma															
No	263	1.00 (reference)	53	1.00 (reference)		66	1.00 (reference)	13	1.00 (reference)		52	1.00 (reference)	17	1.00 (reference)	
Yes	25	0.72 (0.42–1.24)	8	1.13 (0.49–2.64)	0.32	6	0.66 (0.26–1.66)	2	NC	NC	5	0.69 (0.26–1.84)	3	NC	NC
Allergy															
No	85	1.00 (reference)	23	1.00 (reference)		26	1.00 (reference)	7	1.00 (reference)		22	1.00 (reference)	13	1.00 (reference)	
Yes	147	1.19 (0.82–1.72)	34	1.02 (0.55–1.88)	0.63	43	1.10 (0.63–1.91)	10	1.08 (0.39–2.99)	0.98	40	1.13 (0.63–2.03)	13	0.64 (0.28–1.46)	0.23
Surgeries (total number)															
0–6	30	1.00 (reference)	4	1.00 (reference)		9	1.00 (reference)	0	1.00 (reference)		3	1.00 (reference)	2	1.00 (reference)	
≥7	258	1.66 (0.99–2.76)	57	NC	NC	63	1.71 (0.75–3.87)	15	NC	NC	54	**3.56 (1.03–12.2)**	18	NC	NC
Transfusion															
No	446	1.00 (reference)	91	1.00 (reference)		116	1.00 (reference)	24	1.00 (reference)		104	1.00 (reference)	36	1.00 (reference)	
Yes	76	0.78 (0.57–1.09)	27	1.49 (0.91–2.46)	0.01	24	0.95 (0.58–1.57)	8	1.95 (0.81–4.66)	0.14	15	0.68 (0.38–1.24)	10	1.34 (0.62–2.91)	0.12
Birth order															
First/Middle	175	1.00 (reference)	32	1.00 (reference)		41	1.00 (reference)	4	1.00 (reference)		32	1.00 (reference)	15	1.00 (reference)	
Last	67	1.31 (0.87–1.99)	17	1.87 (0.93–3.73)	0.32	19	1.60 (0.85–3.03)	8	NC	NC	21	**2.19 (1.16–4.13)**	3	NC	NC
*Anthropometrics and diet*															
BMI (kg/m2)															
<25	164	1.00 (reference)	42	1.00 (reference)		46	1.00 (reference)	6	1.00 (reference)		39	1.00 (reference)	20	1.00 (reference)	
25–<35	274	0.86 (0.65–1.13)	62	0.73 (0.47–1.14)		63	0.73 (0.47–1.13)	22	1.93 (0.75–4.93)		64	0.89 (0.57–1.40)	21	0.57 (0.30–1.10)	
≥35	48	1.00 (0.64–1.57)	12	0.89 (0.43–1.85)	0.59	18	1.39 (0.73–2.64)	3	NC	NC	5	0.40 (0.15–1.08)	5	0.74 (0.26–2.13)	0.90
Height (in.)															
<65	123	1.00 (reference)	27	1.00 (reference)		33	1.00 (reference)	6	1.00 (reference)		23	1.00 (reference)	13	1.00 (reference)	
65–70	243	0.98 (0.70–1.39)	51	1.06 (0.60–1.88)		58	0.99 (0.58–1.72)	11	0.77 (0.23–2.57)		59	1.48 (0.83–2.64)	19	0.91 (0.40–2.04)	
≥71	125	0.99 (0.62–1.56)	38	1.78 (0.83–3.80)	0.12	38	1.39 (0.68–2.87)	14	1.76 (0.42–7.34)	0.51	26	1.45 (0.66–3.18)	14	1.99 (0.64–6.20)	0.70
Smoking status															
Never	92	1.00 (reference)	22	1.00 (reference)		29	1.00 (reference)	6	1.00 (reference)		26	1.00 (reference)	9	1.00 (reference)	
Ever	108	0.97 (0.65–1.43)	30	1.07 (0.56–2.02)	0.77	29	0.79 (0.43–1.43)	9	1.26 (0.41–3.90)	0.45	24	0.79 (0.42–1.49)	16	1.44 (0.59–3.56)	0.25
Ethanol (g/wk)															
<1	105	1.00 (reference)	26	1.00 (reference)		31	1.00 (reference)	7	1.00 (reference)		27	1.00 (reference)	15	1.00 (reference)	
≥1	93	**0.47 (0.31–0.72)**	27	0.53 (0.28–1.01)	0.73	27	**0.49 (0.26–0.94)**	9	0.54 (0.18–1.68)	0.87	22	**0.37 (0.19–0.74)**	9	**0.24 (0.09–0.63)**	0.43
* Vitamin B6 (mg)*															
<0.97	131	1.00 (reference)	33	1.00 (reference)		41	1.00 (reference)	10	1.00 (reference)		32	1.00 (reference)	12	1.00 (reference)	
≥0.97	67	**0.52 (0.35–0.77)**	20	0.64 (0.34–1.20)	0.52	17	**0.46 (0.24–0.86)**	6	0.72 (0.25–2.10)	0.45	17	**0.52 (0.27–1.00)**	12	1.10 (0.46–2.63)	0.15

**Table 4:** Association (OR and 95% CI) for NHL, DLBCL, and follicular lymphoma for NHL-relevant risk factors (sunlight, environmental exposures), by HLA-DRB1*01:01 allele status and adjusted for age, education, sex, race, and study center.

	All NHL					DLBCL					Follicular				
	Absent		Present		P	Absent		Present		P	Absent		Present		P
	N	OR (95% CI)	N	OR (95% CI)		N	OR (95% CI)	N	OR (95% CI)		N	OR (95% CI)	N	OR (95% CI)	
*Sunlight*															
Sun in teens (h/wk)															
≥14	339	1.00 (reference)	85	1.00 (reference)		51	1.00 (reference)	12	1.00 (reference)		46	1.00 (reference)	22	1.00 (reference)	
<14	122	1.13 (0.74–1.73)	23	0.96 (0.47–1.95)	0.64	18	1.12 (0.60–2.11)	5	1.63 (0.53–5.03)	0.55	15	1.00 (0.51–1.96)	4	NC	NC
Sun in past 10 y (h/wk)															
≥14	173	1.00 (reference)	55	1.00 (reference)		29	1.00 (reference)	14	1.00 (reference)		19	1.00 (reference)	9	1.00 (reference)	
<14	291	1.14 (0.78–1.68)	52	0.75 (0.40–1.39)	0.18	40	0.93 (0.52–1.67)	3	NC	NC	43	1.55 (0.84–2.89)	17	1.31 (0.54–3.18)	0.74
Eye color															
Brown	153	1.00 (reference)	37	1.00 (reference)		23	1.00 (reference)	8	1.00 (reference)		15	1.00 (reference)	9	1.00 (reference)	
Hazel	83	0.98 (0.58–1.65)	15	0.45 (0.17–1.23)		10	0.79 (0.34–1.83)	1	NC		11	1.30 (0.55–3.10)	3	NC	
Green/blue	230	0.88 (0.58–1.34)	56	0.76 (0.39–1.45)	0.74	36	0.91 (0.49–1.67)	8	0.62 (0.22–1.79)	NC	36	1.56 (0.79–3.05)	14	1.07 (0.44–2.64)	NC
*Environmental exposures*															
Termite treatment <1988															
Not treated <1988	339	1.00 (reference)	94	1.00 (reference)		89	1.00 (reference)	25	1.00 (reference)		79	1.00 (reference)	38	1.00 (reference)	
None or DK	82	1.17 (0.82–1.66)	15	0.90 (0.48–1.67)		23	1.24 (0.72–2.15)	4	NC		22	1.29 (0.74–2.25)	6	0.83 (0.33–2.11)	
≥1	102	**1.42 (1.01–1.99)**	10	0.64 (0.31–1.31)	0.02	28	**1.74 (1.04–2.91)**	3	NC	NC	19	1.16 (0.65–2.08)	3	NC	NC
a-Chlordane (dust; ng/g)															
<60.3	227	1.00 (reference)	53	1.00 (reference)		59	1.00 (reference)	14	1.00 (reference)		54	1.00 (reference)	19	1.00 (reference)	
60.3–5,870	71	1.21 (0.80–1.83)	12	1.16 (0.56–2.42)	0.9	17	1.18 (0.85–1.64)	4	NC	NC	15	1.09 (0.78–1.53)	5	1.14 (0.66–1.96)	0.88
PCB180 (dust; ng/g)															
0–20.7	217	1.00 (reference)	48	1.00 (reference)		58	1.00 (reference)	14	1.00 (reference)		52	1.00 (reference)	17	1.00 (reference)	
>20.7	81	1.36 (0.93–1.99)	17	1.25 (0.66–2.38)	0.8	18	1.11 (0.60–2.06)	4	NC	NC	17	1.24 (0.66–2.33)	7	1.71 (0.65–4.48)	0.56
PCB180 (blood; ng/g lipid)															
≤28.7	7	1.00 (reference)	5	1.00 (reference)		2	1.00 (reference)	0	1.00 (reference)		2	1.00 (reference)	3	1.00 (reference)	
>28.7	65	**3.93 (1.49–10.35)**	10	0.66 (0.18–2.37)	**0.02**	9	1.61 (0.33–8.00)	1	NC	NC	12	2.31 (0.47–11.29)	6	NC	NC
Total furans (blood; pg/g lipid)															
≤0.057	8	1.00 (reference)	4	1.00 (reference)		0	1.00 (reference)	0	1.00 (reference)		3	1.00 (reference)	3	1.00 (reference)	
>0.057	64	**2.63 (1.03–6.72)**	11	NC	NC	11	NA	1	NC	NC	11	1.09 (0.27–4.36)	6	NC	NC

Abbreviations: CI, confidence intervals; DLBCL, diffuse large B-cell lymphoma; HLA, human leukocyte antigen; NC, not calculated due to n<5 in cell; NHL, non-Hodgkin lymphoma; OR, odds ratio.

For NHL, statistically significant interactions (p = 0.02) were observed between HLA-DRB1*01:01 and termite treatment before 1988 and blood-based exposures to PCB180 whereby previously reported 1.5-fold elevation in NHL risk for these environmental exposures were limited and statistically significantly only among individuals who did *not* have the HLA-DRB1*01:01 allele. There were no statistically significant interactions specific to DLBCL or follicular lymphoma, though sample size was limited.

### Effects stratified by AH 8.1 (Tables 5 and 6)

**Table 5:** Association (OR and 95% CI) for NHL, DLBCL, and follicular lymphoma for NHL-relevant risk factors (family/medical history and anthropometrics/diet), by AH 8.1 status (adjusted for age, education, sex, race, and study center).

	All NHL					DLBCL					Follicular				
	Absent		Present		P	Absent		Present		P	Absent		Present		P
	N	OR (95% CI)	N	OR (95% CI)		N	OR (95% CI)	N	OR (95% CI)		N	OR (95% CI)	N	OR (95% CI)	
*Family and medical history*															
Family history of NHL															
No	499	1.00 (reference)	81	1.00 (reference)		132	1.00 (reference)	29	1.00 (reference)		130	1.00 (reference)	19	1.00 (reference)	
Yes	22	1.15 (0.62–2.13)	2	NC	NC	5	1.04 (0.38–2.85)	0	NC	NC	7	1.43 (0.58–3.49)	0	NC	NC
Autoimmune conditions															
No	502	1.00 (reference)	79	1.00 (reference)		133	1.00 (reference)	24	1.00 (reference)		130	1.00 (reference)	19	1.00 (reference)	
Yes	26	1.00 (0.58–1.74)	6	1.50 (0.6–3.78)	0.4	6	0.82 (0.33–2.05)	5	**3.85 (1.31–11.3)**	0.02	7	1.07 (0.45–2.55)	1	NC	NC
Asthma															
No	266	1.00 (reference)	38	1.00 (reference)		68	1.00 (reference)	9	1.00 (reference)		60	1.00 (reference)	7	1.00 (reference)	
Yes	23	0.64 (0.37–1.12)	4	NC	NC	6	0.61 (0.25–1.52)	1	NC	NC	3	NC	3	NC	NC
Allergy															
No	91	1.00 (reference)	12	1.00 (reference)		27	1.00 (reference)	5	1.00 (reference)		28	1.00 (reference)	4	1.00 (reference)	
Yes	142	1.09 (0.75–1.59)	31	**2.11 (1.01–4.4)**	0.08	38	0.97 (0.54–1.72)	14	2.26 (0.75–6.84)	0.16	44	1.02 (0.59–1.77)	6	NC	NC
Surgeries (total number)															
0–6	28	1.00 (reference)	2	1.00 (reference)		7	1.00 (reference)	2	1.00 (reference)		3	1.00 (reference)	0	1.00 (reference)	
≥7	261	1.80 (1.07–3.05)	40	NC	NC	67	1.89 (0.82–4.38)	8	NC	NC	60	**3.72 (1.08–12.8)**	10	NC	NC
Transfusion															
No	443	1.00 (reference)	65	1.00 (reference)		113	1.00 (reference)	22	1.00 (reference)		116	1.00 (reference)	15	1.00 (reference)	
Yes	82	0.88 (0.63–1.21)	19	1.38 (0.78–2.45)	0.12	26	1.12 (0.69–1.84)	6	1.20 (0.46–3.15)	0.91	19	0.80 (0.47–1.39)	5	1.63 (0.55–4.80)	0.24
Birth order															
First/Middle	168	1.00 (reference)	25	1.00 (reference)		38	1.00 (reference)	5	1.00 (reference)		39	1.00 (reference)	5	1.00 (reference)	
Last	71	1.40 (0.92–2.13)	11	1.61 (0.73–3.55)	0.73	24	1.92 (1.06–3.49)	3	NC	NC	20	1.78 (0.93–3.42)	4	NC	NC
*Anthropometrics and diet*															
BMI (kg/m2)															
<25	171	1.00 (reference)	25	1.00 (reference)		42	1.00 (reference)	9	1.00 (reference)		48	1.00 (reference)	7	1.00 (reference)	
25–<35	280	0.84 (0.64–1.11)	42	0.79 (0.46–1.36)		75	0.99 (0.64–1.53)	8	0.41 (0.15–1.09)		69	0.80 (0.52–1.22)	12	0.81 (0.30–2.16)	
≥35	42	0.87 (0.54–1.40)	13	1.79 (0.84–3.82)	0.18	12	1.12 (0.54–2.32)	8	**2.96 (1.05–8.29)**	0.25	8	0.58 (0.25–1.32)	0	NC	NC
Height (in.)															
<65	125	1.00 (reference)	12	1.00 (reference)		34	1.00 (reference)	4	1.00 (reference)		29	1.00 (reference)	3	1.00 (reference)	
65–70	241	0.99 (0.70–1.40)	42	2.05 (0.97–4.31)		55	0.90 (0.52–1.57)	12	1.87 (0.53–6.67)		65	1.31 (0.76–2.25)	8	1.73 (0.41–7.42)	
≥71	132	1.03 (0.65–1.63)	26	**2.64 (1.04–6.71)**	0.07	42	1.38 (0.67–2.86)	9	3.05 (0.64–14.6)	0.4	31	1.39 (0.67–2.91)	8	4.17 (0.68–25.5)	0.23
Smoking status															
Never	93	1.00 (reference)	18	1.00 (reference)		27	1.00 (reference)	8	1.00 (reference)		29	1.00 (reference)	5	1.00 (reference)	
Ever	109	0.95 (0.64–1.41)	21	0.84 (0.41–1.73)	0.74	29	0.89 (0.49–1.64)	7	0.53 (0.18–1.59)	0.39	31	0.92 (0.51–1.67)	5	0.62 (0.17–2.33)	0.58
Ethanol (g/wk)															
<1	106	1.00 (reference)	21	1.00 (reference)		29	1.00 (reference)	9	1.00 (reference)		34	1.00 (reference)	5	1.00 (reference)	
≥1	96	**0.50 (0.33–0.75)**	17	**0.41 (0.20–0.88)**	0.63	28	0.51 (0.27–0.99)	6	0.34 (0.1–1.13)	0.52	25	0.30 (0.15–0.59)	4	NC	NC
Vitamin B6 (mg)															
<0.97	130	1.00 (reference)	27	1.00 (reference)		39	1.00 (reference)	11	1.00 (reference)		35	1.00 (reference)	7	1.00 (reference)	
≥0.97	72	**0.56 (0.38–0.84)**	11	**0.41 (0.19–0.89)**	0.43	18	0.49 (0.26–0.93)	4	NC	NC	24	0.69 (0.38–1.25)	2	NC	NC

**Table 6:** Association (OR and 95% CI) for NHL, DLBCL, and follicular lymphoma for NHL-relevant risk factors (sunlight, environmental exposures), by AH 8.1 status (adjusted for age, education, sex, race, and study center).

	All NHL					DLBCL					Follicular				
	Absent		Present		P	Absent		Present		P	Absent		Present		P
	N	OR (95% CI)	N	OR (95% CI)		N	OR (95% CI)	N	OR (95% CI)		N	OR (95% CI)	N	OR (95% CI)	
*Sunlight*															
Sun in teens (h/wk)															
≥14	358	1.00 (reference)	51	1.00 (reference)		49	1.00 (reference)	12	1.00 (reference)		57	1.00 (reference)	8	1.00 (reference)	
<14	116	1.03 (0.67–1.58)	19	1.14 (0.51–2.56)	0.8	16	1.09 (0.56–2.10)	7	2.33 (0.82–6.69)	0.2	15	0.85 (0.44–1.64)	2	NC	NC
Sun in past 10 y (h/wk)															
≥14	202	1.00 (reference)	20	1.00 (reference)		35	1.00 (reference)	7	1.00 (reference)		26	1.00 (reference)	2	1.00 (reference)	
<14	273	0.91 (0.62–1.34)	50	**2.13 (1.00–4.56)**	0.03	30	0.54 (0.29–0.98)	12	1.80 (0.62–5.25)	0.04	46	1.21(0.68–2.15)	8	NC	NC
Eye color															
Brown	167	1.00 (reference)	16	1.00 (reference)		29	1.00 (reference)	2	1.00 (reference)		20	1.00 (reference)	3	1.00 (reference)	
Hazel	83	0.76 (0.45–1.31)	13	1.46 (0.52–4.13)		7	0.38 (0.15–0.97)	4	NC		13	1.16 (0.52–2.60)	1	NC	
Green/blue	228	0.71 (0.46–1.07)	41	1.76 (0.76–4.09)	0.03	29	0.54 (0.29–1.01)	13	NC	NC	39	1.26 (0.68–2.36)	6	NC	NC
*Environmental exposures*															
Termite treatment <1988															
Not treated <1988	347	1.00 (reference)	63	1.00 (reference)		86	1.00 (reference)	23	1.00 (reference)		94	1.00 (reference)	14	1.00 (reference)	
None or DK	84	1.16 (0.81–1.65)	10	0.85 (0.41–1.77)		25	1.31 (0.77–2.25)	2	NC		24	1.18 (0.69–2.02)	3	NC	
≥1	95	1.34 (0.95–1.90)	11	0.97 (0.47–2.00)	0.29	28	1.80 (1.07–3.03)	3	NC	NC	19	1.01 (0.57–1.79)	3	NC	NC
a-Chlordane (dust; ng/g)															
<60.3	231	1.00 (reference)	35	1.00 (reference)		60	1.00 (reference)	12	1.00 (reference)		59	1.00 (reference)	7	1.00 (reference)	
60.3–5,870	62	1.03 (0.83–1.27)	17	**1.49 (1.04–2.12)**	0.04	16	1.08 (0.79–1.48)	3	NC	NC	15	1.02 (0.74–1.40)	5	1.73 (0.95–3.13)	0.11
PCB180 (dust; ng/g)															
0–20.7	215	1.00 (reference)	39	1.00 (reference)		58	1.00 (reference)	12	1.00 (reference)		55	1.00 (reference)	9	1.00 (reference)	
>20.7	78	1.37 (0.92–2.02)	13	1.22 (0.60–2.48)	0.75	18	1.17 (0.63–2.16)	3	NC	NC	19	1.20 (0.66–2.17)	3	NC	NC
PCB180 (blood; ng/g lipid)															
≤28.7	7	1.00 (reference)	4	1.00 (reference)		1	1.00 (reference)	1	1.00 (reference)		2	1.00 (reference)	2	1.00 (reference)	
>28.7	58	3.51 (1.31–9.39)	14	NC	NC	6	1.70 (0.18–16.07)	3	NC	NC	15	2.82 (0.59–13.54)	3	NC	NC
Total furans (blood; pg/g lipid)															
≤0.057	9	1.00 (reference)	3	1.00 (reference)		0	1.00 (reference)	0	1.00 (reference)		4	1.00 (reference)	2	1.00 (reference)	
>0.057	56	1.89 (0.77–4.62)	15	NC	NC	7	NC	4	NC	NC	13	1.02 (0.29–3.54)	3	NC	NC

Abbreviations: CI, confidence intervals; DLBCL, diffuse large B-cell lymphoma; HLA, human leukocyte antigen; NC, not calculated due to n<5 in cell; NHL, non-Hodgkin lymphoma; OR, odds ratio.

We observed statistically significant interactions (p = 0.03) for NHL risk between sun exposure in the past 10 years, eye color and AH 8.1, where the previously reported two-fold NHL risk increase associated with lower sun exposure was restricted to and statistically significant only for individuals *with* the AH 8.1. The interaction (but not the risk estimate) between exposure to sun in the past 10 years remained statistically significant (p = 0.04) for DLBCL. The association between sun exposure in the past 10 years and NHL and DLBCL among individuals *without* the AH 8.1 was null.

The previously reported risk associations between autoimmune conditions and BMI with DLBCL and between height and alpha-chlordane exposure with NHL were all statistically significant only among those with AH 8.1. Additionally, an association between self-reported allergies and NHL was also statistically significant only among those *with* AH 8.1. Among those without AH 8.1, associations for these NHL risk factors were null. There were no statistically significant interactions for follicular lymphoma.

## Discussion

This exploratory evaluation of gene-environment interactions for a broad spectrum of NHL risk factors with implicated NHL risk loci, HLA-DRB1*01:01 and *AH 8.1*, suggests that environmental exposures may interact with HLA-DRB1*01:01 and sun-related exposures with AH 8.1 in altering NHL risk. Additionally, previously reported effects for autoimmune conditions and BMI with DLBCL also appeared restricted among those with AH 8.1. For sufficient power to confirm these observations, especially by NHL subtype, large consortial efforts will be required. Our observation that both HLA-DRB1*01:01 and AH 8.1 are associated with family history of NHL among the control participants offers the first evidence that *HLA* associations may explain some of the well-established relationship between family history and NHL risk [13]. Although this association may seem like an obvious one, our data provide the first empirical evidence for this association and, importantly, previously implicated single nucleotide polymorphisms (e.g., *TNF* and interleukin 10) with NHL have not in fact been shown to be associated with family history of NHL. Other associations between HLA-DRB1*01:01 and AH 8.1 with NHL risk factors among our control population with transfusion history, vitamin B6 intake and sun exposure, support the need for further research in delineating the interrelatedness versus independence of gene and environmental risk factors in understanding lymphoma etiology.

Confirming these potential interactions between NHL risk factors with HLA-DRB1*01:01 and AH 8.1 offers important clues regarding potential mechanisms of action for the implicated risk factors. Interaction with HLA-DRB1*01:01 would implicate autoantibody production in triggering responses to antigens. On the other hand, AH 8.1 is thought to reflect synergistic effects between *TNF* and *HLA* which induces elevated TNF expression and inflammatory responses. Our results thus suggest that NHL risk factors that interact with AH 8.1 (e.g., sun exposure and BMI) might involve inflammatory mechanisms. Recreational sun exposure has been linked to decreased NHL risk in a number of studies and pooled analysis of case-control studies [17], but the mechanism behind this association is presently unknown and proposed mechanisms such as benefits from vitamin D have not been consistently supported in cohort studies [24]. Our previous analyses of gene-environment interactions and sun exposure did not reveal interactions with two candidate immune genes (*TNF* and *IRF4*) [25], [26] but our results here, which demonstrate interaction between AH 8.1 with sun exposure support the hypothesis that sun exposure may modulate NHL risk through altered immunity and inflammation. In addition, components of the AH 8.1, such as HLA-B*08, are associated with a number of autoimmune conditions and NHL risk factors, including systemic lupus erythematosis and Sjogren syndrome. Further investigation of potentially common mechanisms between these risk factors with NHL is thus warranted.

Study strengths include our systematic approach to evaluating the joint effects of two important gene variations with a wide range of NHL risk factors observed in the present study that have been replicated in large pooled analyses or are consistent with the literature [8]. Study limitations include the small sample sizes for some analyses that resulted from the split-sample design of the parent study, decreasing our statistical power to detect significant interactions, particularly within subtypes. In our data interpretation, we assessed previously reported associations to determine whether these known associations were consistent by HLA DRB1*01:01 or AH 8.1 status, or if they were restricted and statistically significant by genotype or haplotype strata. Although we present p-interactions, we did not place sole emphasis on this statistic as they could easily be influenced by risk estimates that lean in opposite directions. Finally, we acknowledge that we may have failed to detect true interactions due to limited statistical power, imperfect measures of exposures and of genes, particularly as not all cis-SNPs necessarily effect local genes and we cannot rule out effects from a trans gene.

Our results require replication in a large independent or pooled effort, such as within the InterLymph Consortium, and among prospective studies. In the emerging view of complex etiologies for NHL with potentially multiple paths to lymphomagenesis, *HLA* associations with NHL may explain a portion of the reported associations between family history and NHL risk. If confirmed in independent data, they provide important evidence that NHL risk factors, including environmental exposures to organochlorines, sun exposure/pigmentation, autoimmune conditions, and BMI, may vary according to a person's HLA-DRB1*01:01 or *AH 8.1* status.
